# History vs. legend: Retracing invasion and spread of *Oxalis pes-caprae* L. in Europe and the Mediterranean area

**DOI:** 10.1371/journal.pone.0190237

**Published:** 2017-12-29

**Authors:** Alessio Papini, Maria Adele Signorini, Bruno Foggi, Enrico Della Giovampaola, Luca Ongaro, Laura Vivona, Ugo Santosuosso, Corrado Tani, Piero Bruschi

**Affiliations:** 1 University of Florence, Dept. Biology (BIO), Florence, Italy; 2 Istituto Agronomico per l’Oltremare (IAO), Florence, Italy; 3 University of Florence, Dept. of AgriFood Production and Environmental Sciences (DISPAA), Florence, Italy; 4 University of Florence, Dept. of Clinical and experimental Medicine (DMSC), Florence, Italy; University of Minnesota, UNITED STATES

## Abstract

*Oxalis pes-caprae* L. is a South African geophyte that behaves as an invasive in the eurimediterranean area. According to a long-established hypothesis, *O*. *pes-caprae* may have invaded Europe and the Mediterranean area starting from a single plant introduced in the Botanical Garden of Malta at the beginning of the 19^th^ century. The aim of this work was to test this hypothesis, to track the arrival of *O*. *pes-caprae* in different countries of the Euro-Mediterranean area and to understand the pathways of spreading and particularly its starting point(s). Historical data attesting the presence of the plant in the whole Euro-Mediterranean region were collected from different sources: herbarium specimens, Floras and other botanical papers, plant lists of gardens, catalogs of plant nurseries and plant dealers. First records of the plant (both cultivated and wild) for each Territorial Unit (3rd level of NUTS) were selected and used to draw up a diachronic map and an animated graphic. Both documents clearly show that oldest records are scattered throughout the whole area, proving that the plant arrived in Europe and in the Mediterranean region more times independently and that its spreading started in different times from several different centers of invasion. Botanical gardens and other public or private gardens, nurseries and plant dealers, and above all seaside towns and harbors seemingly played a strategic role as a source of either intentional and unintentional introduction or spread. A geographic profiling analysis was performed to analyse the data. We used also techniques (Silhouette, Kmeans and Voronoi tessellation) capable of verifying the presence of more than one independent clusters of data on the basis of their geographical distribution. Microsatellites were employed for a preliminary analysis of genetic variation in the Mediterranean. Even if the sampling was insufficient, particularly among the populations of the original area, our data supported three main groups of populations, one of them corresponding to the central group of populations identified by GP analysis, and the other two corresponding, respectively, to the western and the eastern cluster of data. The most probable areas of origin of the invasion in the three clusters of observations are characterized by the presence of localities where the invasive plant was cultivated, with the exception of the Iberian cluster of observation where the observations in the field predate the data about known cultivation localities. Alternative possible reasons are also suggested, to explain the current prevalence of pentaploid short-styled plants in the Euro-Mediterranean area.

## Introduction

The South African tristylous geophyte *Oxalis pes-caprae* L. (Oxalidaceae), native of the Cape Region, is currently a well-known invasive weed in many countries with Mediterranean or even sub-tropical climate [1–8]. Tristily consists in the presence of three different flower morphotypes in relation to the length of the style relatively to the anthers, that is long-styled (L-morph), mid-styled (M-morph) and short-styled (S-morph), accompanied by a diallelic incompatibility system preventing self- and intra-morph fertilization. The three morphotypes can occur simultaneously in the populations or, in other cases, only one or two of the forms may be present [7–10]. In its native area, populations are mainly tetraploid (2*n* = 4*x* = 28), but different ploidy levels are also known, namely diploid (2*n* = 2*x* = 14) and pentaploid (2*n* = 5x = 35), with tristily [7, 9]. In invaded areas, short-styled sterile pentaploids appear to be dominant [7, 9], but tetraploid and monomorphic (L-morph or S-morph), dimorphic (both L-morph and S-morph present) and trimorphic populations have been reported as well, lacking only the monomorphic M-morph type [4, 7, 10–13]. In South Africa the plant grows both in undisturbed sites and as a weed and no clear relation could be observed between style morph or ploidy level and weediness [7]; in native area, reproduction is both sexual by seeds and asexual by bulbils [14]. In invaded regions the plants reproduce basically by bulbils, but in recent years sexual reproduction was also observed in Western Mediterranean region [11–13, 15].

*Oxalis pes-caprae* was introduced in Europe and the Mediterranean region in the 1700s. Starting from the following century, it has become naturalized in that area and subsequently also in several countries of different continents.

Henslow [16] was the first to suppose that all plants currently growing wild in the Euro-Mediterranean area could derive from a single specimen planted in the island of Malta at the beginning of the 19^th^ century by Padre Giacinto, a friar and botanist who founded the Botanical garden of La Valletta. After Henslow, many authors endorsed this hypothesis [e. g.: 1, 17–18]. According to it, all Euro-Mediterranean populations of *O*. *pes-caprae* possibly would have derived from a single individual or a small population: this could also explain why pentaploid short-styled plants are dominant—if not exclusive—of the area. The presence of dimorphic and trimorphic populations in the mediterranean may be explained only by genetic rearrangement of the founder individual,

Yet, is this hypothesis correct? The aim of this study was to verify it by means of available original documents, in order to: 1) trace the arrival of *O*. *pes-caprae* in different countries of the Euro-Mediterranean area; 2) indicate possible means and pathways of spreading and its starting point(s).

Geographic Profiling (GP) is an analytic method first introduced by Rossmo [19] to identify the geometrical origin of crimes by a serial killer. This method was later applied to other cases in which a series of linked events could be mapped on a geographical chart, such as the origin of a biological invasion [20–22], for the prediction of nest location of bumble bees [23] or of an epidemy [24–25]. GP analyzes a series of map coordinates of the occurrence of linked events to create a geoprofile, that is a probability surface [19] on the map itself. Such geoprofiles will allow to prioritize geographical points [19], so helping in individuating the most probable areas from which the events originated. Recently further techniques have been coupled with geoprofiling, allowing to analyze if the data are more probably derived from a single point of origin or more than one, that is Kmeans an algorithm for partitioning data [26], Silhouette [27] for assessing the right number of clusters and Voronoi tessellation for partitioning the territory on which data are collected [28].

Phylogeographical methods by the use of DNA markers variation, such as microsatellites have been used for reconstruction of biological invasions and their introduction and spreading pathways in plant species [29–30]. The technique allows to link the marker variation with the geographical occurrence of populations belonging to a given species. Ferrero et al. [31] investigated the genetic diversity in 10 and 12 populations from South Africa and the Western Mediterranean region, with nuclear microsatellite loci. To check if also the plastid genetic variation corresponded to the nulear data by Ferrero et al. [31], we performed a preliminary analysis of plastidial DNA markers (microsatellites) variation in *O*. *pes-caprae* in the Euro-Mediterranean area.

## Materials and methods

### Exsiccata and literature check

Data sources since the first records up to 2010 were the following:

1. Herbarium specimens. Over 50 important European herbaria were contacted. Information, images or herbarium specimens were obtained from 28 of them (ANC, BC, BM, BR, CAG, CAT, CLU, FI, FIAF, G, GDOR, GE, K, L, LEC, LISU, NAP, NICE, P, PAD, PI, RO, SIENA, TO, UTV, W, WAG, WU).
2. Published records. These data included: floristic papers: national and local Floras; other floristic contributions; vegetational and other geobotanical papers; any other scientific contribution dealing with the species (systematic, spread, agricultural impact) and including distributional data; lists of plants grown in Botanical Gardens and in other public or private gardens; catalogs of plant nurseries and/or plant dealers (see references in S1 Table).
3. Field observations.

Direct observations on the fields were mainly restricted to italian populations, as confirmations of previous reports. Some new observation from an historical point of view and not present in literature, were those about the presence by the Botanical Garden of Catania in 1887 and by the Botanical Garden of Perugia (beginning of the 20^th^ century). The samples were collected by hand in year 2010. All the collected information was filed in a database or Analytical Table (recorded as a spreadsheet). From this, a Synthetic Table was derived, including only the earliest records for each Territorial Unit (TU), corresponding to differently named administrative units (counties, provinces or others), according to the country (see supplementary material S2 Table). For European Countries and Turkey, TU were intended at the 3rd level of NUTS (Nomenclature of Units for Territorial Statistics) [32]. For non-European countries, data have been taken from the GADM database of Global Administrative Areas [33], using the more detailed level available. Records of cultivated and wild plants were separately dealt with.

The material used directly by the authors do not require specific permissions of collection, since they are sample of a well known invasive and surely not endangered species.

### Molecular data

We performed a preliminary phylogeographical analysis on the basis of chloroplasts microsatellites data obtained from a sampling of 37 populations for a total of 399 individuals of *O*. *pes-caprae*. For each individual we collected 2–5 leaves that were conserved at -80°C. The 37 sampling localities are reported in Table 1.

**Table 1:** Distribution of the five haplotypes (chloroplast) in the sampled populations.

	HAPLOTYPE 122–121			HAPLOTYPE 121–122			HAPLOTYPE 120–119		
	POPULATION	%	PLOIDY LEVEL (from [9])	POPULATION	%	PLOIDY LEVEL (from [9])	POPULATION	%	PLOIDY LEVEL (from [9])
1	Cyprus	100,00%		Castiglioncello (IT)	20,00%		Capoliveri (IT)	8,33%	2n = 35
2	Lisbon	100,00%		Chiessi (IT)	6,66%		Malta2	16,66%	
3	Piombino (IT)	100,00%		Rodi Garganico (IT)	6,66%	2n = 35	Chiessi (IT)	13,33%	
4	Giglio C. (IT)	90,00%		Hanbury (IT)	40,00%		Rodi Garganico (IT)	6,66%	2n = 35
5	Giglio A. (IT)	100,00%		Malta1	37,50%		Stintino (IT)	6,66%	2n = 35
6	Calabria1 (IT)	100,00%	2n = 35	Rio Marina (IT)	13,33%		Malta3	6,66%	
7	Genova O. B. (IT)	63,63%	2n = 35	Giglio C. (IT)	10,00%		Pianosa (IT)	7,69%	2n = 35
8	Sestri (IT)	70,00%		Genova O. B. (IT)	36,36%	2n = 35	SUM =	65,99%	
9	Imperia (IT)	76,92%	2n = 35	Sestri (IT)	30,00%		MEAN =	1,78%	
10	Ittiri (IT)	100,00%	2n = 35	Imperia (IT)	7,69%	2n = 35			
11	Palermo O.B. (IT)	90,00%		Palermo O. B. (IT)	10,00%	2n = 35	HAPLOTYPE 122–120		
12	Ficarra (IT)	100,00%	2n = 35	Capoliveri (IT)	8,33%	2n = 35	POPULATION	%	PLOIDY LEVEL (from [9])
13	Capoliveri (IT)	75,00%	2n = 35	Cavo (IT)	10,00%	2n = 35	Imperia (IT)	7,69%	2n = 35
14	Capraia (IT)	100,00%		Malta2	16,66%		Tanqua Karoo (SA)	33,33%	2n = 28
15	Cavo (IT)	80,00%	2n = 35	SUM =	253,19%		Chiessi (IT)	6,66%	
16	Enfola (IT)	100,00%		MEAN =	6,84%		Hanbury	20,00%	
17	M. Campo-Lac. (IT)	100,00%	2n = 35				Marocco	10,00%	2n = 35
18	Malta2	66,66%		HAPLOTYPE 121–120			SUM =	77,68%	
19	S. Andrea (IT)	80,00%	2n = 35	POPULATION	%	PLOIDY LEVEL (from [9])	MEAN =	2,09%	
20	Kirstenbosch (SA)	100,00%	2n = 28, 35	Imperia (IT)	7,69%	2n = 35			
21	Tanqua Karoo (SA)	66,66%	2n = 28	Capoliveri (IT)	8,33%	2n = 35			
22	Jacobsbaii (SA)	100,00%	2n = 28 (56)	Cavo (IT)	10,00%	2n = 35			
23	Calabria2 (IT)	100,00%	2n = 35	S. Andrea (IT)	20,00%	2n = 35			
24	Castiglioncello (IT)	70,00%		Castiglioncello (IT)	10,00%				
25	Chiessi (IT)	66,66%		Chiessi (IT)	6,66%				
26	Rodi Garganico (IT)	80,00%	2n = 35	Rodi Garganico (IT)	6,66%	2n = 35			
27	Stintino (IT)	86,66%	2n = 35	Stintino (IT)	6,66%	2n = 35			
28	Giannutri (IT)	100,00%		Rio Marina (IT)	13,33%				
29	Hanbury (IT)	40,00%		SUM =	89,33%				
30	Malta3	93,33%		MEAN =	2,41%				
31	Malta1	62,50%							
32	Marocco	90,00%	2n = 35						
33	Pianosa (IT)	92,30%	2n = 35						
34	Pollina (IT)	100,00%	2n = 35						
35	Rio Marina (IT)	73,33%	2n = 35						
36	Riomaggiore (IT)	100,00%	2n = 35						
37	Ventotene (IT)	100,00%							
	SUM =	3213,65%							
	MEAN =	86,85%							

The 37 sampling localities are reported with the known ploidy level in the respective populations (if known). IT = Italy; SA = South Africa.

The used markers were located on the pastidial genome. Since the plastidial inheritance is uniparental in most angiosperms, the use of the plastome for phylogeographical analysis should be complimentary with respect to the biparentally inherited nuclear genome [34].

We extracted total genomic DNA from 100 mg leaf fragments with EZNA kit (Eazy Nucleic Acid Isolation Omega), on 96 wells plates, following the manufacturer protocol. Quantity and quality of DNA was checked with agarose gel electrophresis. We amplified 6 universal plastidial microsatellites and summarized the respective position of the loci in the plastome in supplementary material as S3 Table, the repeated sequences at each locus, the sequences of the primers used for amplifying the loci, the original annealing temperature and the dimension of the microsatellite in tobacco as in Weising and Gardner [35].

PCR reaction was executed in a total volume of 12.5 μL containing 20–25 ng (3 μL) of DNA template, 1U (0.125 μL) of Taq polymerase (Promega), 2.5 μL 5X buffer containing 1.5mM MgCl_2_ (Promega); 0.25 μL (0.1 μM) for both forward and reverse primers; 0.25 μL DNTPs (10 μM); 0.125 μL BSA; 6 μL H_2_O. The amplification program comprised an initial denaturation step at 94°C for 5 minute, 30 cycles of 30 s at 94°C, 30 s at 55°C annealing temperature and 60 s at 72°C. Finally we used an extension step of 7 minute at 72°C, followed by cooling at 4°C. The separation of the DNA fragments was executed with an AB 3130xl genetic analyzer (Applied Biosystems). After the evaluation of the qualifty of amplified fragments and of the polymorphism present, we chose two primer pairs for two loci: ccmp3 and ccmp4 (as defined by Weising and Gerdner [35]).

The results were summerized in a matrix, later analysed considering the polymorphism at the 6 loci with Genalex 6.2 [36]. The alleles were grouped to identify the different plastidial haplotypes.

### Geographic profiling

This protocol is described step by step with examples in the site https://www.protocols.io with the dx.doi.org/10.17504/protocols.io.kytcxwn. The observations derived from historical records (the oldest record for each locality) were drawn on the map and converted to a csv file of values with x and y coordinates. Only the first records were used to avoid overweighting of some localities simply due to successive records from the same populations. The series of observations were partitioned in clusters with the Kmeans clustering method [26]. The more probable number of clusters was assessed with the Silhouette method [27]. Afterwards a Geographic profiling analysis was performed on each cluster to assess the invasion origin as proposed by Stevenson et al. [20], Papini et al. [21], Cini et al. [22].

For performing the GP analysis the following software was used (written by Alessio Papini and Ugo Santosuosso): Kmeans_sil_0_0_2.py for assessing the most probable number of clusters with the Silhouette method and Geoprof2_0_5csv.py for the geographic profiling analysis.

Both programs are available online (www.unifi.it/caryologia/PapiniPrograms.html, https://bitbucket.org/ugosnt/al_and_ugo/) and were written and executed in Python 2.7.3 (http://www.python.org/) run on a Linux Ubuntu 12.04 LTS (http://www.ubuntu.com/) Operating System, Linux kernel 2.6.32. The programs require Python (> = 2.6 version) and need NumPy (http://www.numpy.org/), SciPy (http://www.scipy.org/), Matplotlib (http://matplotlib.org/), Scikit-learn (http://scikit-learn.org) and Python Image Library -PIL- (http://www.pythonware.com/products/pil/) libraries installed. All of them are released open source and under GPL distribution licence.

## Results

Over 1200 distribution records were collected and filed into a general Analytical table (not reported).

The derived Synthetic table (see suppl. material S2 Table) includes 181 first reports of the plant in the study area. Later reports for a previously reported location were not inserted in the synthetic table. Data concerning cultivated plants were recorded for 30 TU, those concerning wild plants for 151 TU.

**Fig 1:**
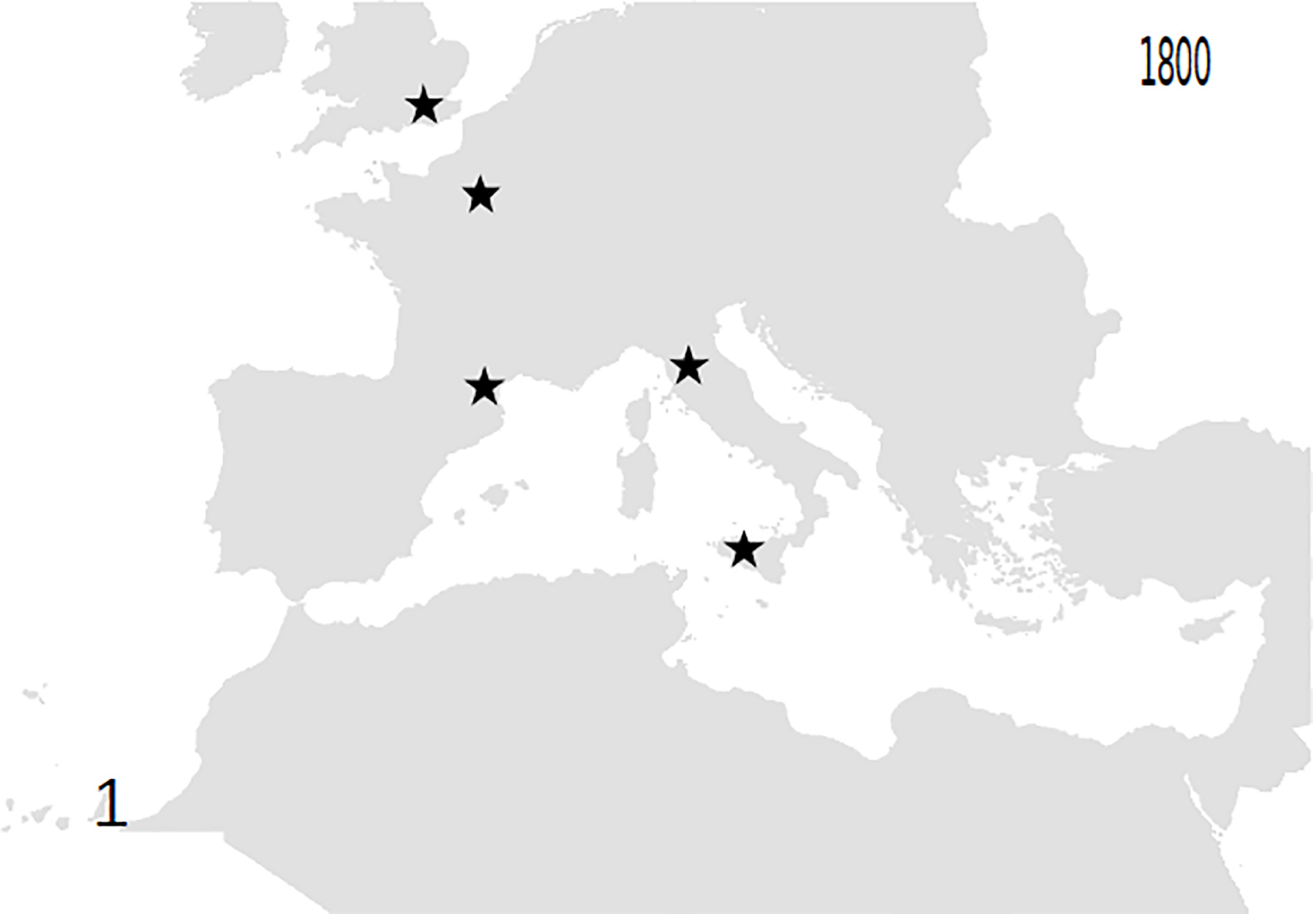
Point of known presence of *O*. *pes-caprae* in the Euro-Mediterranean area until 1800. Only cultivated plants are known.

**Fig 2:**
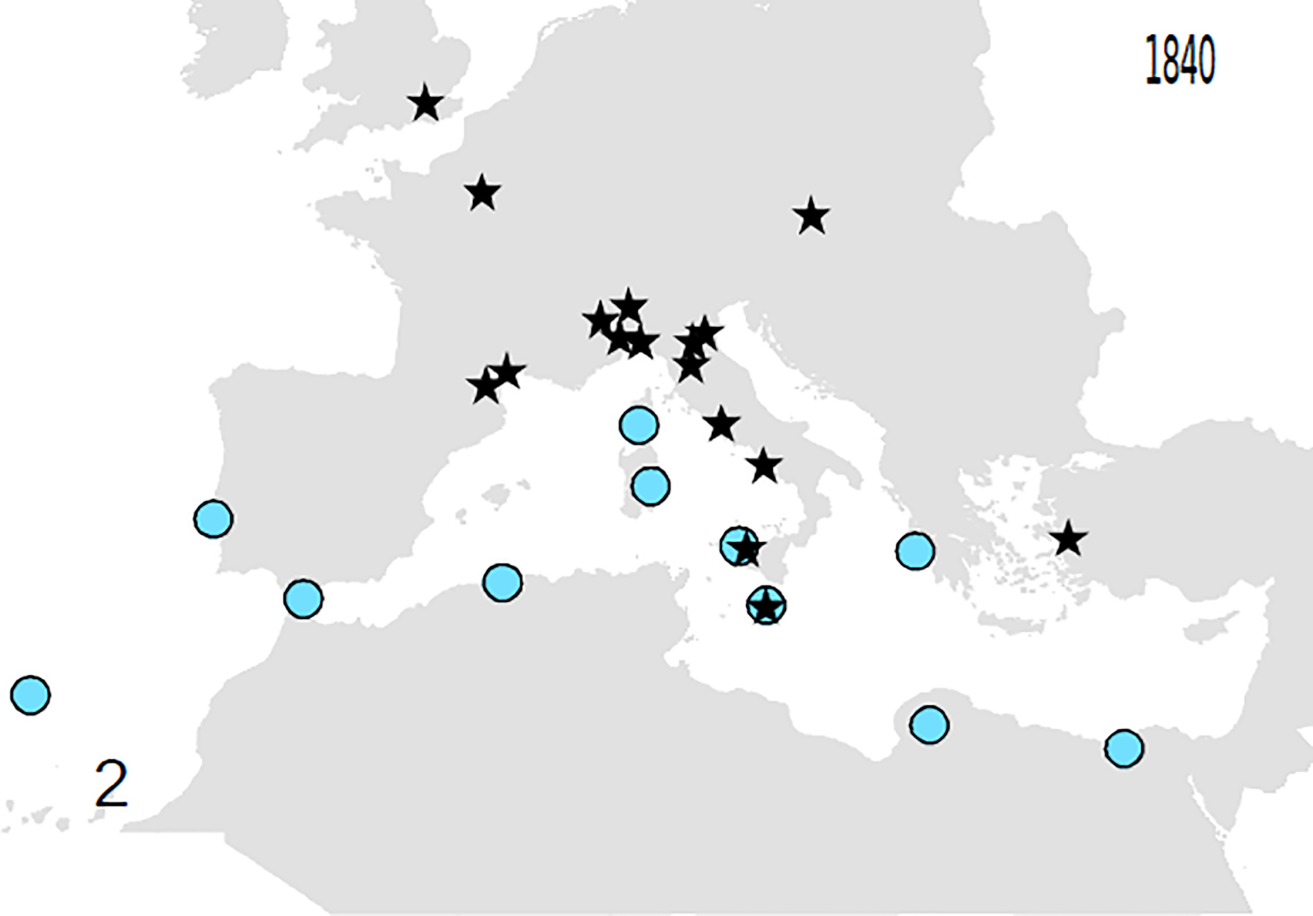
Point of known presence of *O*. *pes-caprae* in the Euro-Mediterranean area until 1840. Indications of the plant in the wild are known in south mediterranean.

**Fig 3:**
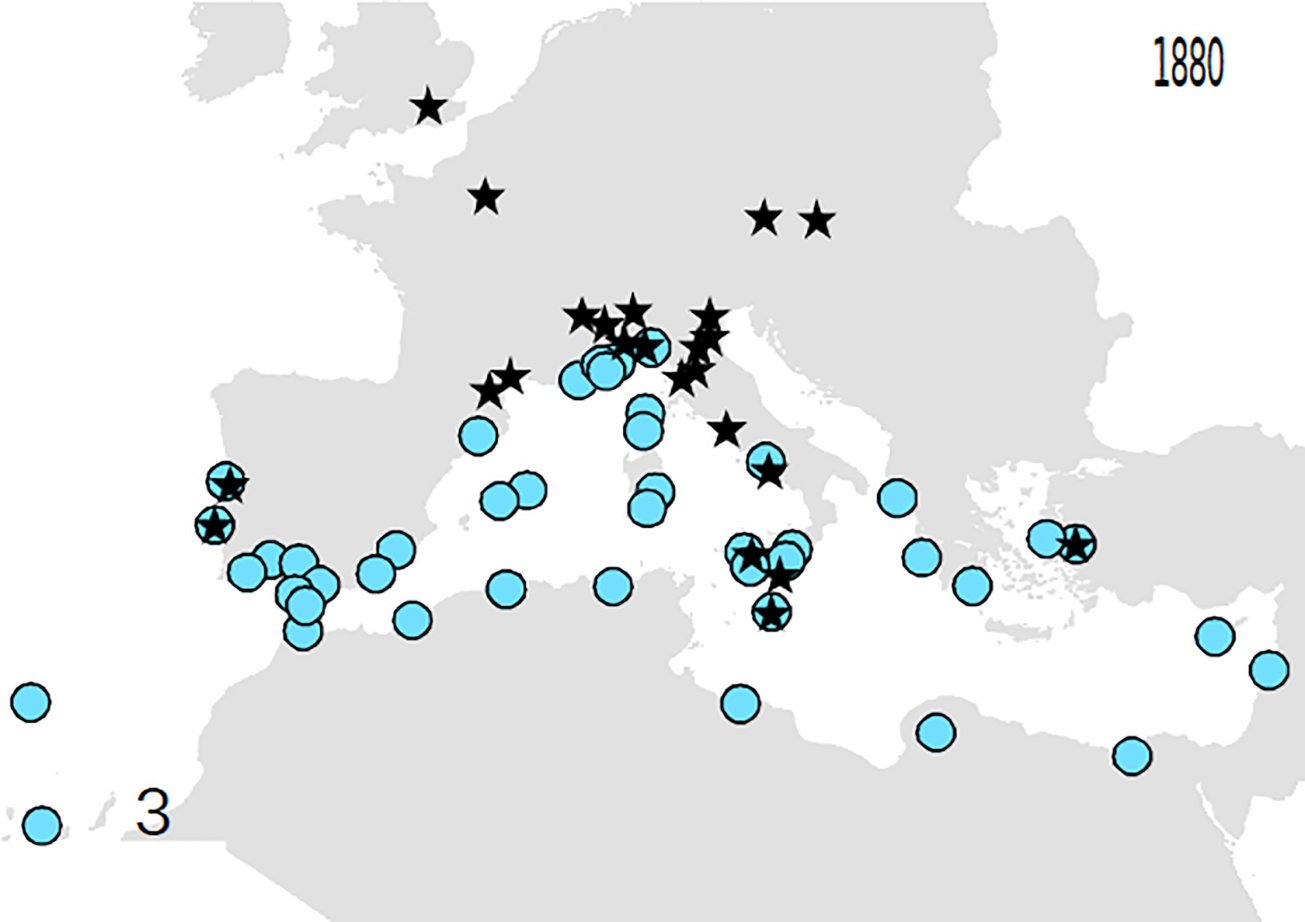
*O*. *pes-caprae* in the Euro-Mediterranean area until 1880. More stations in the wild are known, but always limitedly to south mediterranean.

**Fig 4:**
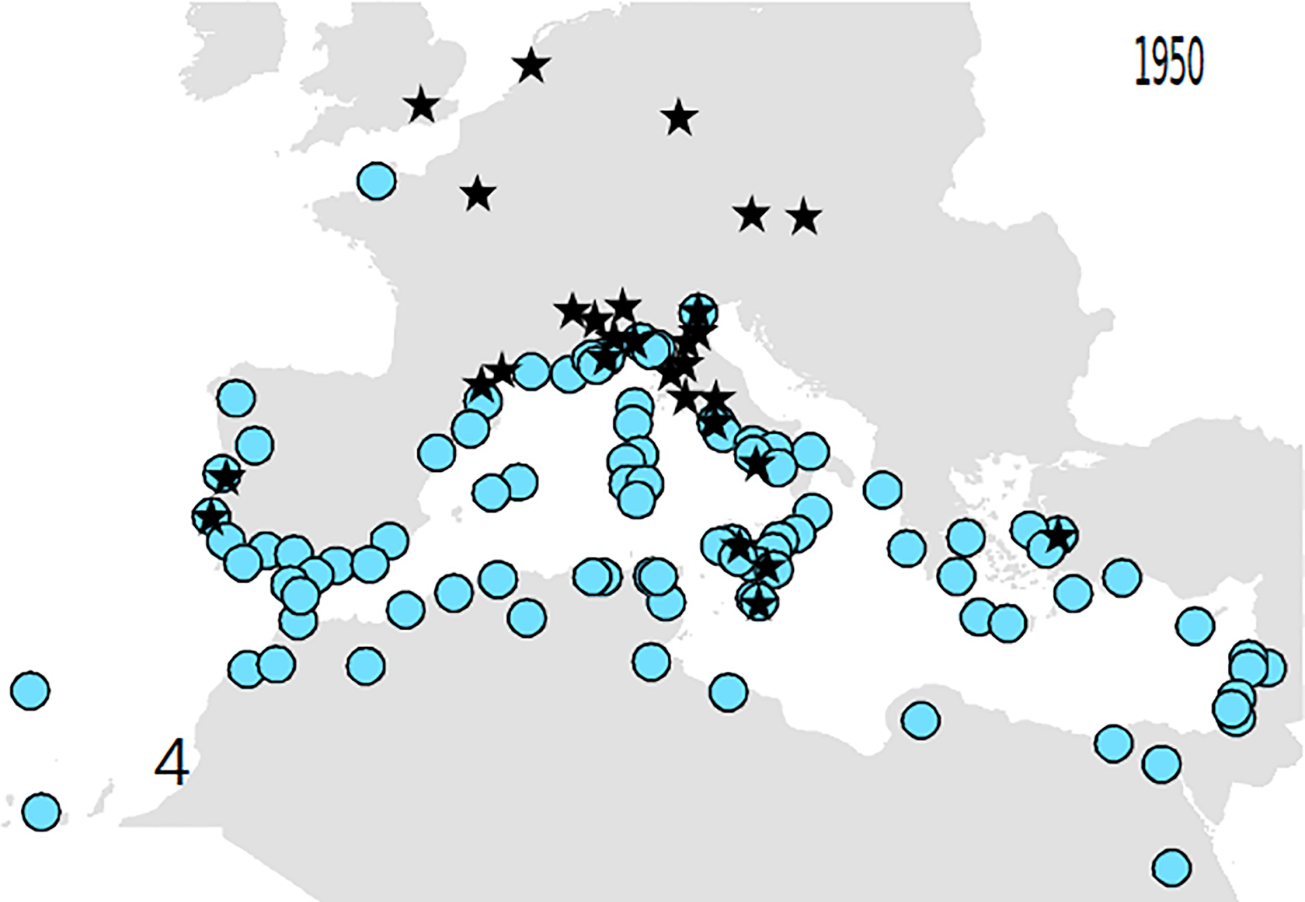
*O*. *pes-caprae* in the Euro-Mediterranean area until 1950. Some stations are indicated in a more northern position. Black stars: records of cultivated specimens. Blue circles: records of wild plants.

**Fig 5:**
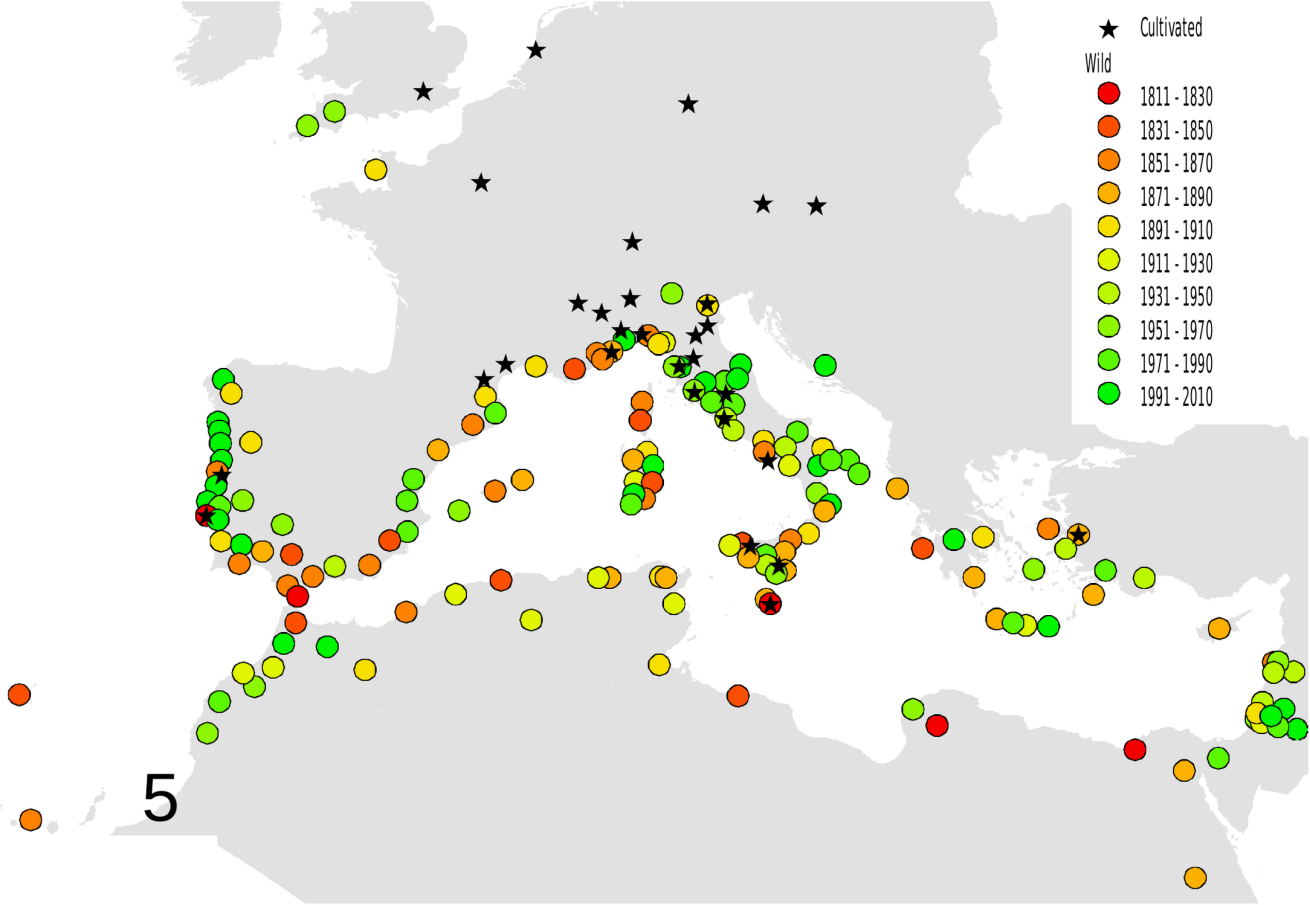
Arrival and spread of *Oxalis pes-caprae* L. General diachronic map of the presence of *O*. *pes-caprae* in the Euro-Mediterranean area (black stars: records of cultivated plants). The legends indicate the correspondence of the different colors of the circles on the map (the observation) with the first year of the record for a given population.

Based on these records, the following outputs were obtained: 1—An animated graphic (in supplementary material as S1 Video), showing the dates and places of earliest reports throughout the decades. From this, single maps can be derived, pertaining to different times (Figs 1–4).; 2—A general diachronic map of the presence of the plant in the Euro-Mediterranean area (Fig 5).

### Molecular data

We used two markers of the tested six. We managed to identify five different haplotypes on the basis of the used markers. The most common haplotypes, A, (122–121) was present in all populations, with a frequency of 89% in the South African populations and 80% in the invasive populations in the mediterranean. 17 populations (two from South Africa and 15 from the Euro-Mediterranean area) resulted monomorphic for this marker. The haplotype E (2.09% frequency) was found in five populations, one of them in South Africa (Tanqua Karoo) in the native area of the species, with a frequency of 33%, one in the examined Moroccan population and three populations a subset of the populations where also haplotype B was found. The haplotype B (121–121), was not found in the samples from South Africa (original area of the species), but it was found in 14 populations in the Euro-Mediterranean area, with an average frequency of 7%. It was mostly represented in four populations: Hanbury garden (40%), Malta 1 (37%), Genova Botanical Garden (36%) and Sestri Levante (30%). This group of observations corresponded to a group of early cultivations in botanical gardens of southern Europe and possibly to one of the two subdivisions identified by GP analysis already in 1840. The haplotypes C (121–120) and D (120–119) were not found in the analyzed South African populations. Haplotype C had a frequency of 2.41% and was present in nine populations, constituting a subset of the populations where also haplotype B was present. Haplotype D (1.78% frequency) was found in seven populations, corresponding in part to a subset of the populations with haplotype B, plus three populations belonging to the same cluster identified by GP. The analysis of the DNA markers were summarized in Table 1 (DNA data).

The phylogeographical analysis on the basis of the microsatellites data, even with the limitations due to the limited sampling in South Africa, showed that the data may be divided into three main clusters with a geographical distribution corresponding to the central cluster identified by GP (characterized by haplotypes B, C, D and E) and other two clusters where haplotype A prevailed, one in western Europe and one in eastern Europe, apparently the most recent one.

### Geographic profiling analysis

**Fig 6:**
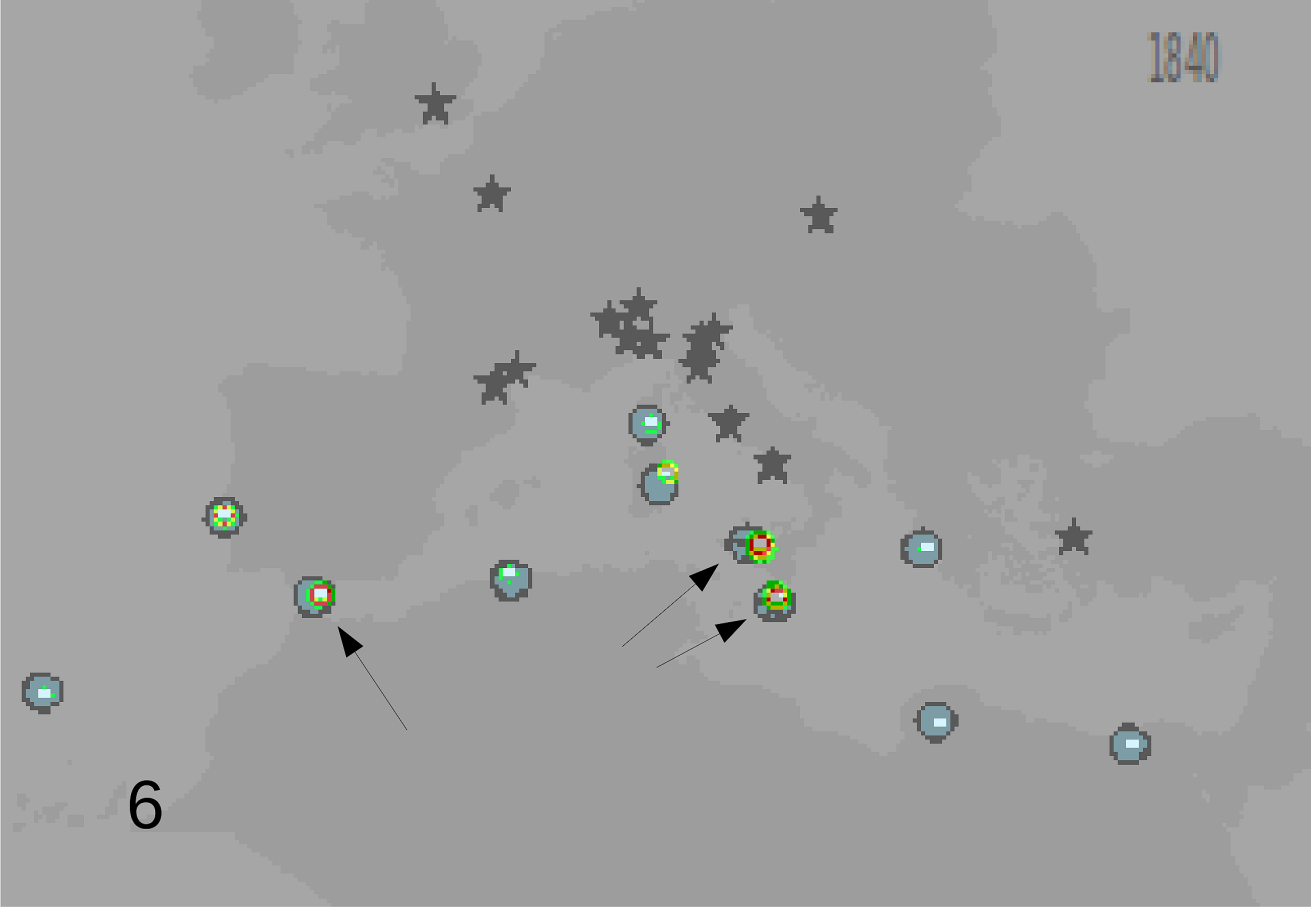
Kmeans and geographic profiling analysis results on the data of 1840. Two clusters were the most probable solution after the Silhouette criterion. The arrows show the most probable spread origin for the first cluster (left arrow) and for the second one (central arrows), this last compatible with the hypothesized origin from Malta. In red the areas of highest probability of the spread origin.

**Fig 7:**
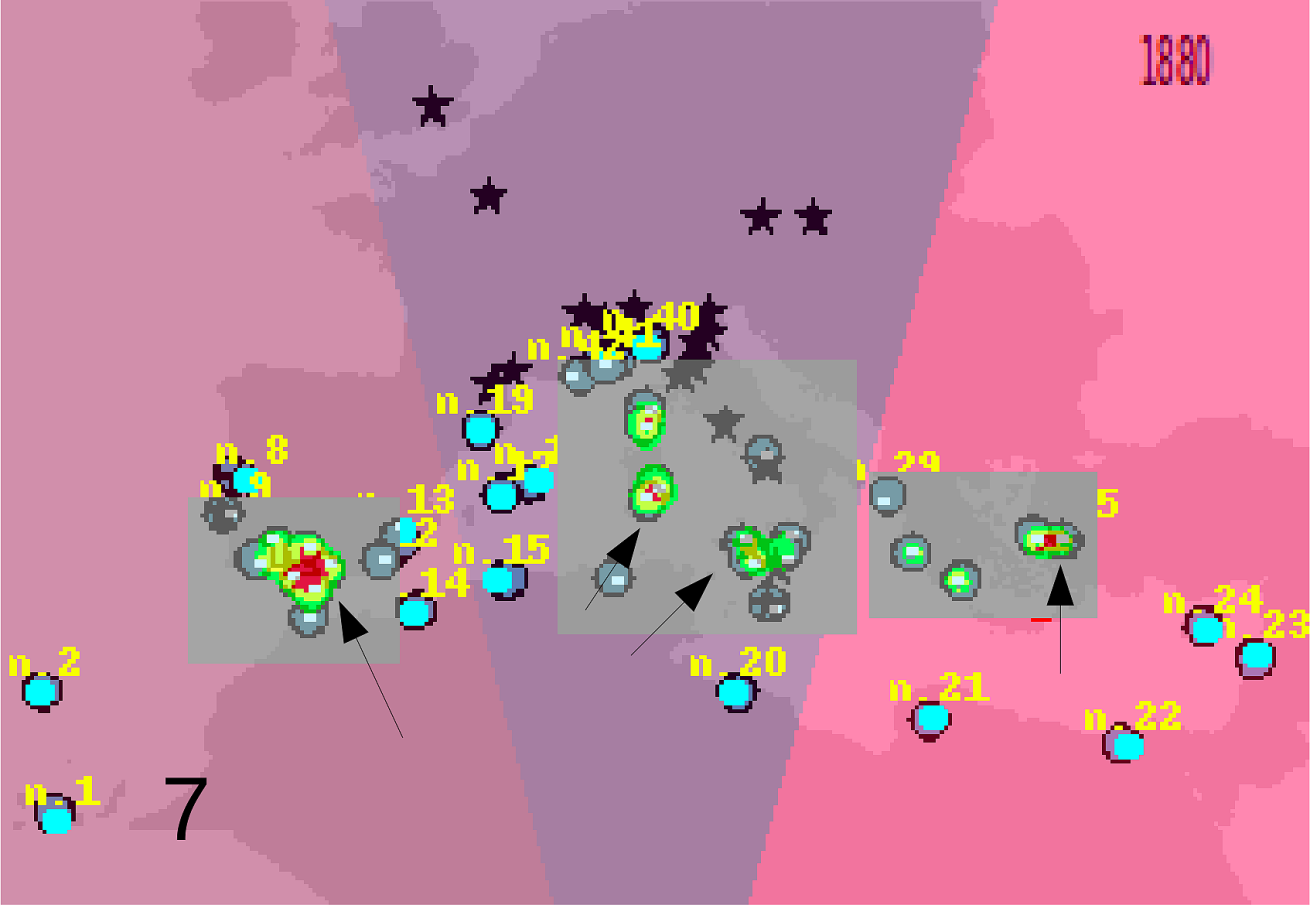
Kmeans and geographic profiling analysis results on the data of 1880. Three clusters were the most probable solution after the Silhouette criterion. The arrows show the most probable spread origin for the first cluster (left arrow), for the second cluster (central arrows), and for the third cluster (western coast of Turkey). In red the areas of highest probability of the spread origin. The numbers represent the observation number in the CSV file containing the geographical data. The Voronoi tessellation on the basis of the three clusters is leared with different colors.

**Fig 8:**
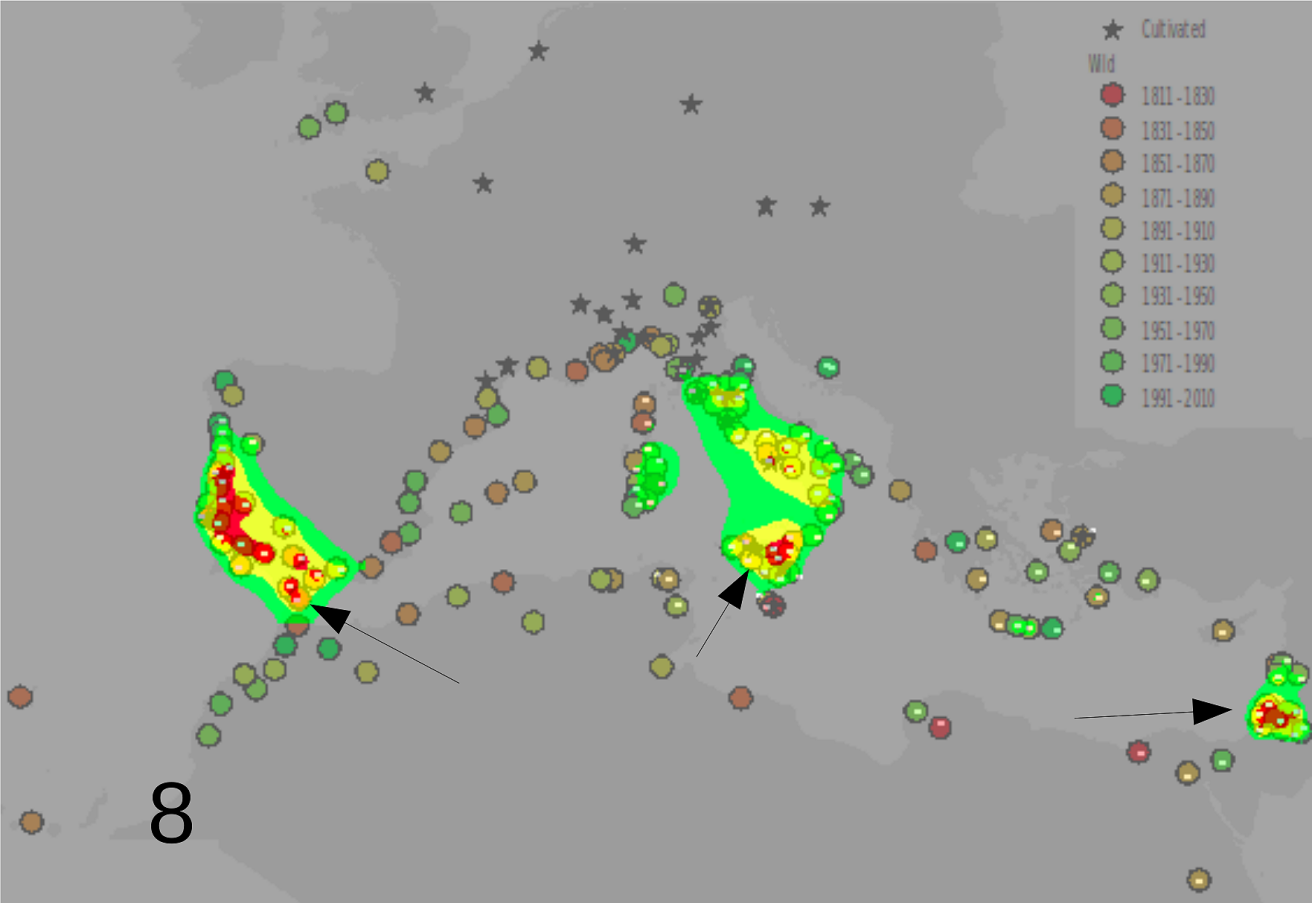
Kmeans and geographic profiling analysis results on the data until 2010. Three clusters were the most probable solution after the Silhouette criterion. The arrows show the most probable spread origin for the clusters, confirming the hypothesis obtained with data until 1880. In red the areas of highest probability of the spread origin. Legend as in Fig 5.

The Silhouette analysis results (see supplementary material: S1 Fig for 1840, S2 Fig for 1880 and S3 Fig for present data) indicated as the more probable number of clusters in which to partition the data, two for the 1840 distribution map (Fig 6) and 3 for the 1880 (Fig 7) and present (until 2010) distribution maps (Fig 8). An origin for each cluster is shown in Fig 6 for 1840 (arrows indicated the most probable origin after the GP analysis, in red the area of highest probability). Data of year 1880, analyzed with GP showed that the most probable number of cluster was three, with three independent centers of origin (Fig 7). For the central cluster the GP analysis indicated two areas of most probable origin of the invasion: one in Sicily/Malta and one about 300 chilometers further north in southern Sardinia (Fig 7). Fig 8 shows the present situation. In this last analysis the higher amount of data confirmed the hypothesis of three centers of origin and reduced the uncertainty in the position of the origin of the central cluster (now only in Sicily), but without changing significanty the results obtained with the more reduced data set of 1880.

## Discussion

The main aims of our investigation were: to trace the arrival of *O*. *pes-caprae* in different countries of the Euro-Mediterranean area and to give a clue about the possible means and pathways of spreading and particularly its starting point(s). The capability of assessing the point of origin of a biological invasion is fundamental for understanding the mode of introduction in general of invasive organisms via common routes (for instance the lessepsian way of invasion in the Mediterranean from the Red sea [21]) and, possibly, to provide suggestions in order to contrast the expansion of the spread [21, 28]. Geographic profiling may produce results useful to determine the the starting point of an invasion, and the results are better as much as it is possible to provide the best possible geographic locations on the map of interest. It must be underlined that processed historical distribution data (both sites and dates) are possibly incomplete and sketchy due to many factors. These factors may include: uneven geographical and chronological distribution of geobotanical and other scientific investigations through past decades and centuries; rough dating (in general, only a *terminus ante quem* can be deduced); records from public and private gardens mostly focused on Italy, due to some difficulties found in getting data from other countries; lack of records of cultivated plants, especially in private spaces, as they often escape scientists’ attention; difficulties in finding some possible kinds of data sources (e.g.: old commercial plant catalogues). Also data based on herbarium samples can be biased. For instance, Delisle et al. [37] pointed out that active sampling periods of herbarium samples are very variable during the time, and a consequence of that is that it is possible to follow exactly the spreading of an invasive species during periods of active herbarium samples collections, while in other period of lower activity speed and modality of the spreading would be understood only partially. This is particularly true for the detection of the point of start of the invasion, when the invading plant would still be rare in the area and probably neglected. Mihulka & Pyšek [38] observed in thei study about spreading species belonging to genus *Oenothera*, that also wrong identification of closely related species may play a role in confounding the general pattern of the invasion. Nevertheless, herbarium and literature data is often the only available information and must be taken in account with the due attention, particularly in geographical regions where collection of samples is more limited as in East Africa [39] and in the tropicas in general.

However, the available data about *O*. *pes-caprae* allow some deductions:

Exsiccata and literature data clearly demonstrate that in the Euro-Mediterranean area, the plant arrived in the past more times independently in different places and times: the ‘monocentric’ Malta hypothesis [1, 16] is definitely not supported by historical records. As a matter of fact, earliest records of wild plants of *Oxalis pes-caprae* appear to be scattered throughout the whole southern part of the considered area, from east to west, without any apparent order. In the first half of the 19^th^ century they were observed in the following places (see suppl. material S2 Table, places given in chronological order): Malta, Cyrenaica (Libya), Gibraltar (Iberian Peninsula, UK), Lisbon (Portugal), Alexandria (Egypt), Tortolì (Sardinia, Italy), Palermo (Sicily, Italy), Zakynthos island (Greece), Ajaccio (Corse, France), Funchal (Portugal), Algeri (Algeria), Seville (Spain), Tangier (Morocco), Tripoli (Libya), Hyères (France), Cartagena (Spain).

From the diachronic map (Fig 5), it also comes out that botanical gardens and other public or private gardens played a strategic role as a source of both intentional and unintentional introduction or spread, where local climatic conditions were suitable for the species. No plants escape was recorded from Botanical Gardens of Kew, Paris, Vienna and even Florence, while evident links between cultivated and naturalized plants can be pointed out in some cases (see S2 Table);

- nurseries and plant dealers, as *Oxalis pes-caprae* was grown and sold as an ornamental plant, but is also a common weed in potted plants (especially *Citrus*), potting soil, garden wastes, fertilizers, etc.
- ports and seaside towns, where plants and/or bulbils possibly arrived together with goods transported by cargo ships.

The GP analysis suggested multiple introductions: two until 1840, becoming three in 1880, with a more eastern cluster of observation. This results would confirm Ferrero et al.’s [15, 31] conclusion, based on SSR nuclear markers, that there has been more than an introduction in the mediterranean area. It is interesting to observe that the highest amount of data of 2010 did not change the results (three clusters with centers of origin, respectively, in southern Spain, southern Sicily/Malta and western coast of Turkey). This fact confirms the high robustness of the data obtained with geographic profiling even with few data and hence at the beginning of a biological invasion. Another interesting point is that all the red areas (areas of most probable first introduction) of each cluster, correspond to areas where it is known the presence of old cultivation of *O*. *pes-caprae*. The only exception appears to be that of the iberian cluster, where the observations in the field apparently predate the known events of cultivation (compare Fig 8 with Figs 2–4). This finding may be useful for future avoidance of further spreading.

The results of the chloroplast microsatellites analysis is not yet exhaustive from the point of view of sampling, particularly in the South African original area of distribution, and from the point of view of the reduced number of markers (unfortunately, only few were polymorphic among those tested). Nevertheless, the phylogeographical analysis, on the basis of microsatellites data, even if with a limited sampling in the native area range of the species and a possibly too low number of markers, was sufficient to show that the data may be divided into three main clusters with a geographical distribution corresponding with the central cluster identified by GP (characterized by presence of haplotypes B, C, D and E) and other two clusters where haplotype A prevailed, one in western Europe and one in eastern Europe. The microsatellites analysis revealed haplotypic diversity particularly also among populations (see Table 1), further supporting the conclusion that a single introduction is not probable, since it would imply a very low genetic variation. Even if our data are from plastidial origin, hence probably with a lower base substitution rate with respect to the nuclear markers employed in [31], it is interesting to observe that plastidial data overlap with the nuclear data. Ferrero et al. [31] proposed that also the occurrence of mixed ploidy levels and polymorphism of the style in Europe is consistent with multiple introductions into the Western Mediterranean. Nevertheless, we have to consider that populations in the Euro-Mediterranean area and particularly in Italy, are almost all pentaploid and hence would spread mainly via vegetative propagation, a mode that would not promote the increase in genetic diversity, even if some seed formation in pentaploids was observed by Ferrero et al. [31], at least in the iberian peninsula. The absence of an haplotype in the South African populations with respect to the European ones is instead probably related to the insufficient sampling of the South African populations in this study.

The phylogeographical analysis based on microsatellites DNA variation hence individuated two clusters of populations with respect to the three identified by GP analysis, one of them corresponding to the cluster appearing in 1840 and corresponding to the central cluster of three identified by GP in Fig 7. The same number of clusters was identified by Ferrero etal. [31]. Since the genetic data provided by nuclear markers do not always overlap with the data obtained from plastidial data, as in the complex case of mediterranean oaks [40, 41], it is meamingful to observe that in Oxalis the nuclear data used in [31] are confirmed by the plastidial ones. It is interesting to observe that all the italian populations and one from Morocco tested until now for their chromosome number and stylar form, resulted all pentaploid and short-styled [9]. This group of populations correspond to the central cluster identified by the Geographic Profiling analysis (see Figs 7 and 8). This data supported the hypothesis by Ferrero et al. [31] of multiple origin, even if these last authors based their analysis mainly on western Europe sampling, and proposed only two main introduction events corresponding to a group of introduced tetraploids and another with prevailingly pentaploids. Interestingly Ferrero et al. [31] analysis was based on nuclear markers. These authors concluded that multiple introductions may explain a genetic diversity in the western Mediterranean populations, that is higher than expected. On the basis of the current data the most recent cluster of *O*. *pes-caprae*, the eastern one of Fig 7, may have derived from a long distance dispersal (in the period 1840–1880 after the GP analysis) from the western cluster, even if the eastern cluster is still underrepresented in the genetic analysis.

Some problems still remain open. If the ‘monocentric’ hypothesis (i.e. a single individual or small population starting the spreading from Malta) is to be discarded, why pentaploid short-styled plants are so prevailing in the Euro-Mediterranean area, and in some regions possibly even exclusive? In order to clear up this phenomenon, further investigations are needed, which will also take into account that in the native area the pentaploid chromosome race is quite uncommon [7, 42] and that pentaploid plants are currently known only with the short-styled morphotype [9–11].

Among possible explanations, the following can be suggested:

- selection during travel from native area: did pentaploid plants (or bulbs) turn out to be more stress-resistant? Pentaploid plants may result to be more resistant against adversities and/or more successful as a weed, as suggested by Baker [5], who considered the pentaploid *O*. *pes-caprae* owning a general-purpose genotype strategy. As a matter of fact, in case of ploidy heterogeneity, polyploids tend to prevail in invaded area possibly by selection of the polyploid [42–44] or, more rarely, by genome duplication [42, 45].
- allochtonous origin of the pentaploid race: its origin could have occurred outside native area, maybe more times independently. The polyploidization in polyploid series within “species” may be a consequence of environmental selection, see, for instance, the increase in polyploidy in *Larrea tridentata* associated with the increased summer aridity [46]. The capability of fast adaptation by polyploids was recognized as a consequence of the increase of possible phenotypes able to survive in habitats outside the original native area [47–49].

Later, invasive pentaploid plants may have possibly been re-introduced in South Africa as an ‘alien’ weed, as suggested by Signorini et al. [50] and Krejčíková et al. [39]. This hypothesis is partially supported by Ferrero et al. [31] genetic data, and would deserve a wider sampling in the native area to be confirmed.

## Conclusions

Our data showed the progressive increase in number of observations of *O*. *pes-caprae* in the mediterranean on the basis of historical (exsiccata and literature) data. The analysis of the possible the partitions of data (observations) with Silhouette, Kmeans and finally with geographic profiling on each cluster of data suggested that three successive introductions may have occurred and provided the most probable areas of the first introduction. GP analysis allows also to suggest that the first two introductions would have occurred earlier than 1840, while the third east-mediterranean one would have occurred between 1840 and 1880. Such results (about multiple introductions) were corroborated by preliminary microsatellites data results based on chloroplast markers and confirmed previous results by other authors based on nuclear markers [31], who found the presence of at least two distinct clusters in Europe (one pentaploid and one tetraploid).

## Supporting information

S1 Table Observation references. The published records are listed. These data include: floristic papers: national and local Floras; other floristic contributions; vegetational and other geobotanical papers; any other scientific contribution dealing with the species (systematic, spread, agricultural impact) and including distributional data; lists of plants grown in Botanical Gardens and in other public or private gardens; catalogs of plant nurseries and/or plant dealers.
(PDF)

S2 Table Synthetic Table of the distribution data. In this table, only the earliest records for each Territorial Unit (TU) are included. Geographical entities described after NUTS (Nomenclature of Units for Territorial Statistics).
(XLS)

S3 Table Plastidial microsatellites. Data on amplification of 6 universal plastidial microsatellites. The position of the loci in the plastome, the repeated sequences at each locus, the sequences of the primers used for amplifying the loci, the original annealing temperature and the dimension of the microsatellite in tobacco as in Weising and Gardner [35] are summarized.
(XLSX)

S1 Video Animated graphic. It shows the dates and places of earliest reports of *O*. *pes-caprae* in the Mediterranean throughout the decades.
(MP4)

S1 Fig Silhouette analysis results for observations up to 1840. The situation with two clusters is the most homogeneous, as an indication of the most probable number of clusters.
(PDF)

S2 Fig Silhouette analysis results for observations up to 1880. The situation with three clusters is the most homogeneous, as an indication of the most probable number of clusters.
(PDF)

S3 Fig Silhouette analysis results for observations up to nowadays (up to 2010). The situation with three clusters is the most homogeneous, as an indication of the most probable number of clusters.
(PDF)
