# Supplementary material for: History vs. legend: Retracing invasion and spread of Oxalis pes-caprae L. in Europe and the Mediterranean area
Source: PLoS One. 2017 Dec 29;12(12):e0190237. doi: 10.1371/journal.pone.0190237 (PMC5747460; doi:10.1371/journal.pone.0190237)
Supplement: S1 Table — The published records are listed. These data include: floristic papers: national and local Floras; other floristic contributions; vegetational and other geobotanical papers; any other scientific contribution dealing with the species (systematic, spread, agricultural impact) and including distributional data; lists of plants grown in Botanical Gardens and in other public or private gardens; catalogs of plant nurseries and/or plant dealers. (PDF) [file pone.0190237.s001.pdf]

1. Aiton W, Bauer FA, Ehret GD, Nicol G, Sowerby J (1789) Hortus Kewensis, or, A catalogue of the plants cultivated in the Royal Botanic Garden at Kew. George Nicol, London

2. Balbis GB (1801) Synopsis plantarum Horti botanici Taurinensis. Torino

3. Ballero M, Cara S, Marras G, Loi MC (2000) La flora del Fluminese (Sardegna sud occidentale). Webbia 55 (1):65-105

4. Barceló y Combis F (1867) Apuntes para una Flora de las Islas Baleares (part I). Madrid

5. Berger A (1912) Hortus Mortolensis: enumeratio plantarum in Horto Mortolensi cultarum. West, Newman, London

6. Bertoloni A (1824) Bononiensis vegetabilia cum aliis vegetalibus commutanda ad annuum 1824. Bologna

7. Bocchieri E, Iiriti G (2003) Flora di Teccu, un promontorio basaltico della Sardegna centro-orientale. Atti Soc Toscana Sci Nat Pisa, Mem, Ser B 110:35-53

8. Bogdanović S, Dobrović I, Ostojić A, Boršić I (2003) Oxalis pes-caprae L., a new species in the Flora of Croatia. Nat Croat 12 (1):31-37

9. Bonnet E, Barratte G (1896) Catalogue raisonné des Plantes vasculaire de la Tunisie, Impr. Nationale, Paris

10. Braun-Blanquet J, Maire R (1924) Études sur la végétation et la flore Marocaines. Comptes-Rendus des Herborisations de la Société Botanique de France, Session du Maroc, 1921. Mem Soc Sci Nat Maroc 8(1):1-244

11. Burollet P A (1927) Le Sahel de Sousse; Monographie Phytogéographique. Société anonime de l'Impr. rapide de Tunis, Tunis.

12. Campana A (1812) Catalogus plantarum Horti Botanici Regii Lycei Ferrariensis. G. Bresciani, Ferrara

13. Castro S, Ferrero V, Costa J, João Sousa A, Castro M, Navarro L, Loureiro J (2013) Reproductive strategy of the invasive Oxalis pes-caprae: distribution patterns of floral morphs, ploidy levels and sexual reproduction. Biol Invasions 15 (8):1863-1875. doi: 10.1007/s10530-013-0414-2

14. Castro S, Loureiro J, Santos C, Ater M, Ayensa G, Navarro L (2007) Distribution of flower morphs, ploidy level and sexual reproduction of the invasive weed Oxalis pes-caprae in the western area of the Mediterranean Region. Ann Bot 99: 507-517

15. Cattaneo A (1812) Catalogo delle piante più interessanti del Giardino Cattaneo per l'anno 1812. Tip. Rasario, Novara

16. Colmeiro y Penido M (1885) Enumeración y revisión de las plantas de la Península Hispano-Lusitana é islas Baleares, con la distribución geográfica de las especies, y sus nombres vulgares,

tanto nacionales como provinciales. Impr. de las Viuda é Hija de Fuentenebro, Madrid

17. De Bolòs O, Vigo J (1990) Flora dels Països Catalans Vol.2. Editorial Barcino, Barcellona.

18. De Candolle AP (1813) Catalogus Plantarum Horti botanici Monspeliensis: addito observationum circa species novas aut non satis cognitas fasciculo. Jardin des Plantes, Montpellier

19. De Marco G, Mossa L (1973) Ricerche floristiche e vegetazionali nell'Isola di San Pietro (Sardegna): la flora. Ann Bot (Rome) 32: 155-215

20. De Visiani R (1857) Semina in Horto Botanico Patavino lecta anno 1857. Orto Botanico, Padova

21. Della Giovampaola E (2010) Oxalis pes-caprae L., specie invasiva nella regione mediterranea: indagini ecologiche, biosistematiche e molecolari. PhD thesis, Dottorato di Ricerca in Biosistematica ed Ecologia Vegetale, Scuola di Dottorato "Ubaldo Montelatici" (XXIII ciclo), Università degli studi di Firenze, Firenze

22. Donarelli C (1836) Selectus stirpium viventium quae in Horto Botanico Romanae Studiorum Universitatis pro mutua commutatione offeruntur anno 1836. Orto Botanico, Roma

23. Ducellier L (1913) L'Oxalis cernua en Algerie. Sa destruction. Imprimerie agricole Montégut, Alger

24. Grech Delicata JCG (1853) Flora Melitensis. F.W.Franz, Malta

25. Haage & Schmidt (1884), Catalogue autumn 1884, Erfurt

26. Hantz J (1986) Distribution of Oxalis pes-caprae L. in the East Mediterranean region. Ann Mus Goulandris 7: 49-56.

27. Henslow G (1894) On the northern distribution of Oxalis cernua Thunb. Proc Linn Soc London 1892-1893: 31-36.

28. Kelaart EF (1846) Flora Calpensis. J. Van Voorst, London

29. Lange J (1860) Pugillus plantarum imprimis hispanicarum, quas in itinere 1851-52 legit Joh. Lange. B. Luno, Copenhagen

30. Marquand ED (1901) Flora of Guernsey and the lesser Channel Islands. Dulau, London

31. Meikle RD (1977) Flora Of Cyprus. Bentham-Moxon Trust, Kew

32. Merino B (1905) Flora descriptiva é ilustrada de Galicia. Tipografía Galaica, Santiago

33. Nègre R (1962) Petite flore des régions arides du Maroc occidental. CNRS, Paris

34. Pangella M (1812) Premier supplement au Catalogue des plantes cultivées dans le jardin de Mr.

De
35. Freylin-Buttigliera a Buttigliera (Marengo). François Pila, Asti

36. Parlatore F (1872) Flora italiana. Le Monnier, Firenze

37. Pignatti S (1982) Flora d'Italia. Edagricole, Bologna

38. Pitard CJM (1913) Exploration scientifique du Maroc organisée par la Société de Géographie de Paris. Premier fascicule: Botanique (1912). Masson et Cie, Paris

39. Post GE (1932) Flora of Syria, Palestine and Sinai. American University of Beirut, Beirut

40. Rappa F (1911) Osservazioni sull'Oxalis cernua. Boll R Orto Bot Giard Colon Palermo 10:142-185

41. Rouy G, Foucaud J (1897) Flore de France. Les Fils d'Émile Deyrolle, Paris.

42. Santos do Vale J de SF (1842) Catalogus Plantarum Academiae Conimbricensis Horti. Manuscript

43. Silipranti G (1885) Contribuzione alla flora dei dintorni di Noto. Atti Soc Nat Modena Mem Ser III, 4:22-44

44. Sommier S (1904) Comunicazione all'adunanza dell'8 maggio 1904 della Società Botanica Italiana (sede di Firenze). Nuovo G Bot Ital Nuova Ser 11:177-178

45. Sommier S, Caruana Gatto A (1915) Flora Melitensis Nova. Stabilimento Pellas, Firenze

46. Tenore T (1813) Catalogus Plantarum Horti regii Neapolitani ad annum 1813. Orto Botanico, Napoli

47. Vaccari A (1899) Flora dell'Arcipelago di Maddalena. Malpighia 13 (nr. 1-4)

48. Van Tubergen CG Ltd. (1932), Catalogue autumn-spring 1932-33, Haarlem

49. Viegi L, Cela Renzoni G, Garbari F (1974) Flora Esotica d'Italia. Lav Soc Ital Biogeogr 4:126-220

50. Viegi L, Cela Renzoni G (1981) Flora esotica d'Italia: le specie presenti in Toscana. Consiglio Nazionale delle Ricerche, Pavia

51. Viegi L, Vangelisti R, D'Eugenio ML, Rizzo AM, Brilli-Cattarini A (2003) Contributo alla conoscenza della Flora esotica d'Italia: le specie presenti nelle Marche. Atti Soc Toscana Sci Nat Mem Ser B 110: 97-162

52. Viviani D (1802) Elenchus plantarum horti botanici J. Cav. Di Negro. Genova

53. Willkomm M, Lange J (1880) Prodromus Florae Hispanicae. E. Schweizerbart, Stuttgart

54. Young DP (1957) Oxalis in the British Isles. Watsonia 4: 51-69

55. Zuccagni A (1782) Synopsis plantarum horti botanici Musei Regii Florentini anno 1782. Orto Botanico, Firenze
