# Supplementary figures and images for: History vs. legend: Retracing invasion and spread of Oxalis pes-caprae L. in Europe and the Mediterranean area
Source: PLoS One. 2017 Dec 29;12(12):e0190237. doi: 10.1371/journal.pone.0190237 (PMC5747460; doi:10.1371/journal.pone.0190237)

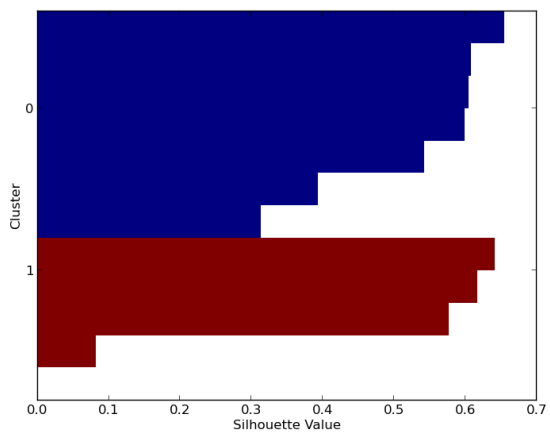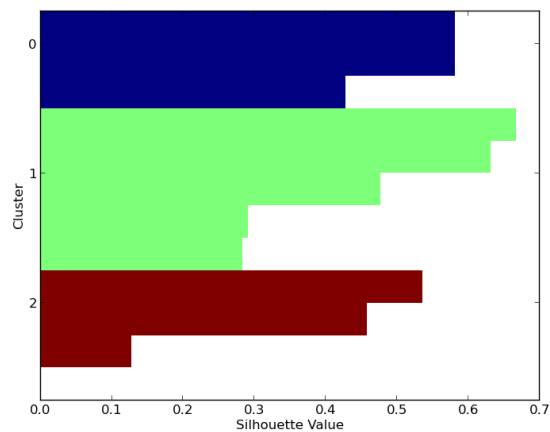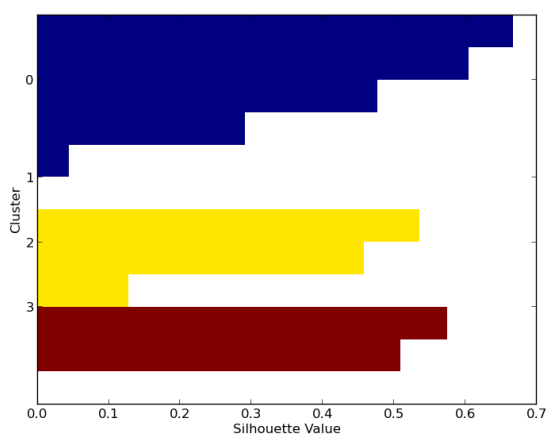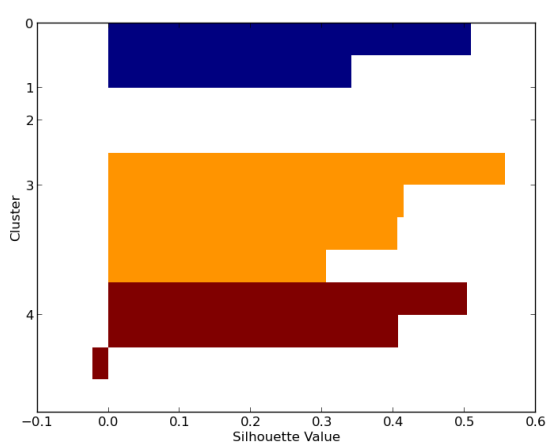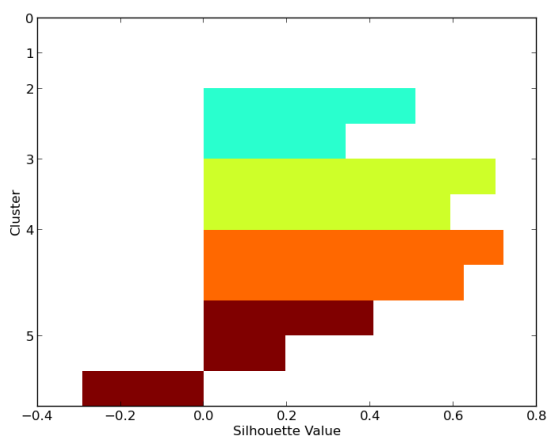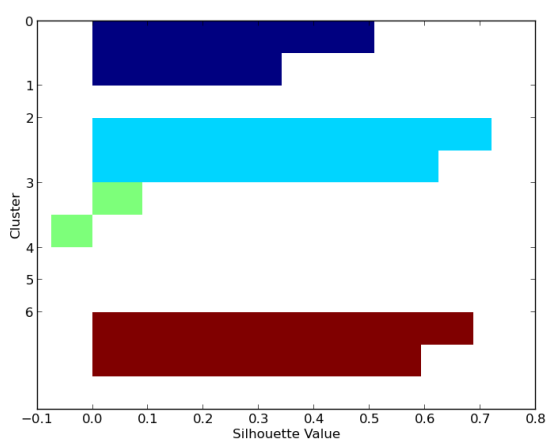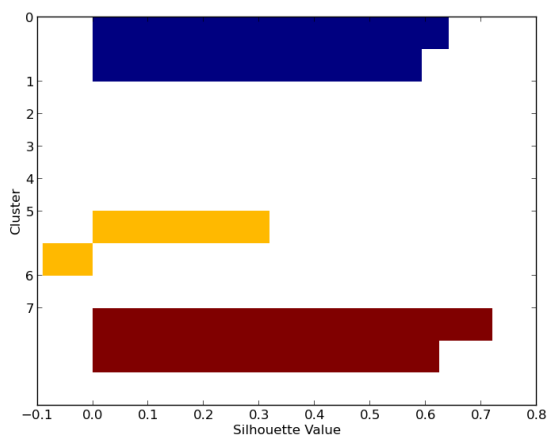

Supplement: S1 Fig — The situation with two clusters is the most homogeneous, as an indication of the most probable number of clusters. (PDF) [file pone.0190237.s005.pdf]

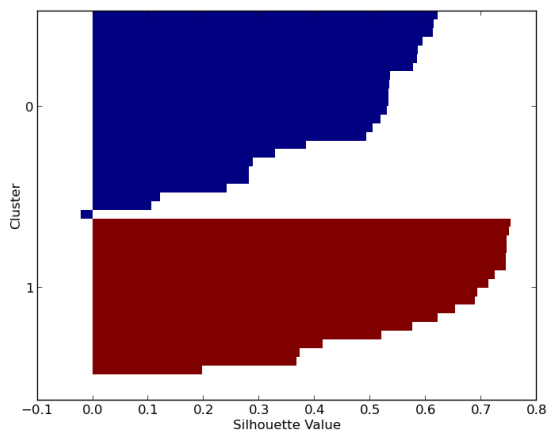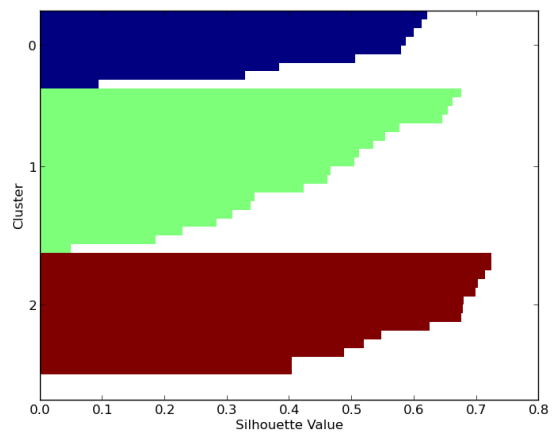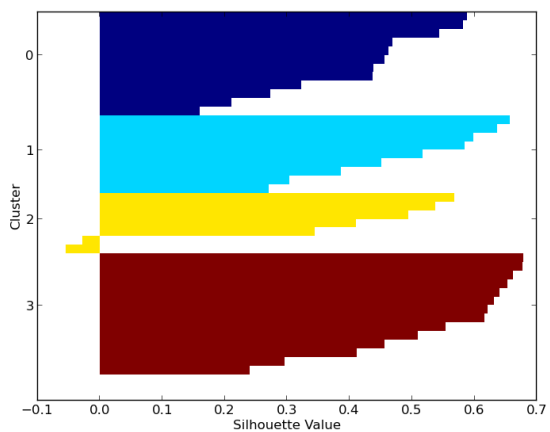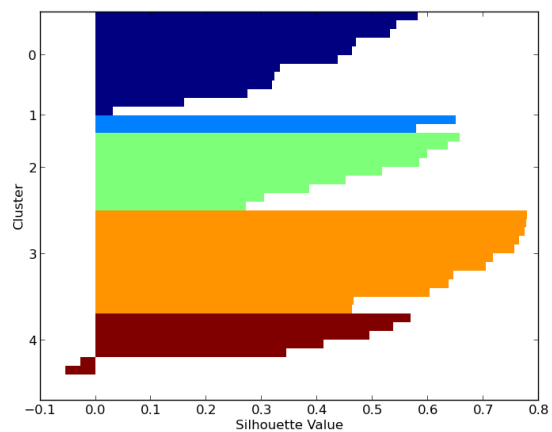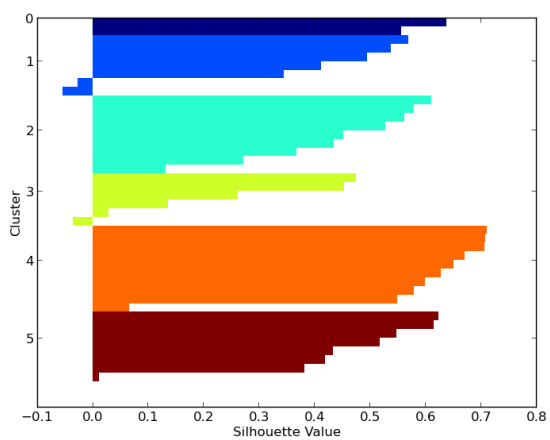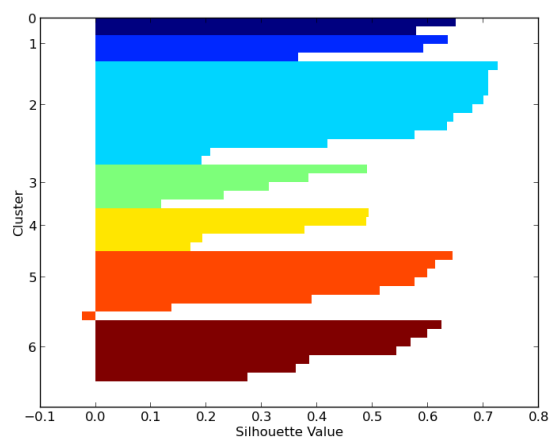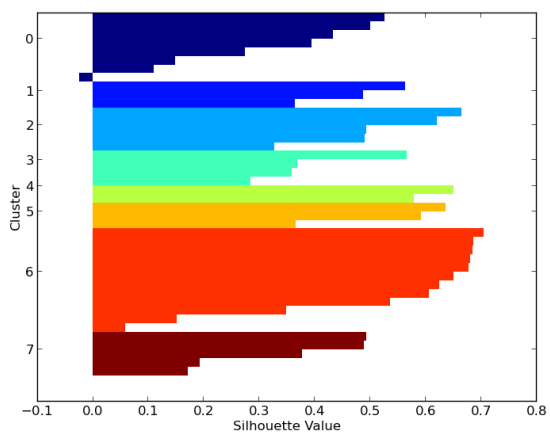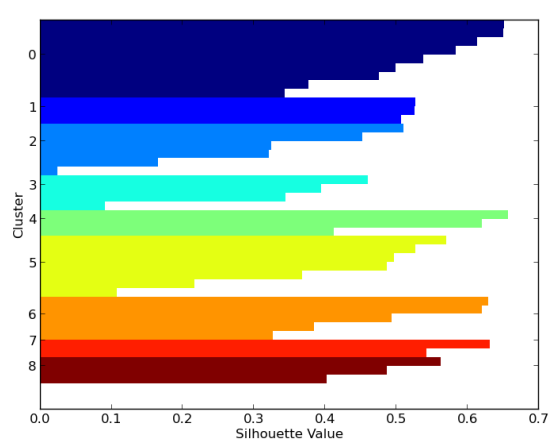

Supplement: S2 Fig — The situation with three clusters is the most homogeneous, as an indication of the most probable number of clusters. (PDF) [file pone.0190237.s006.pdf]

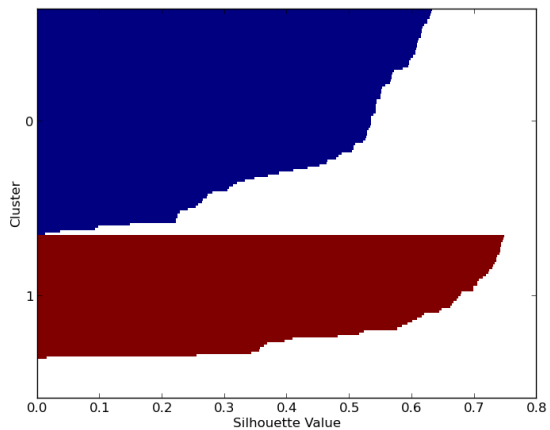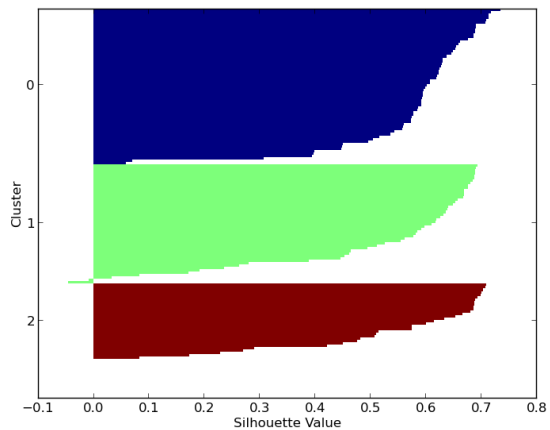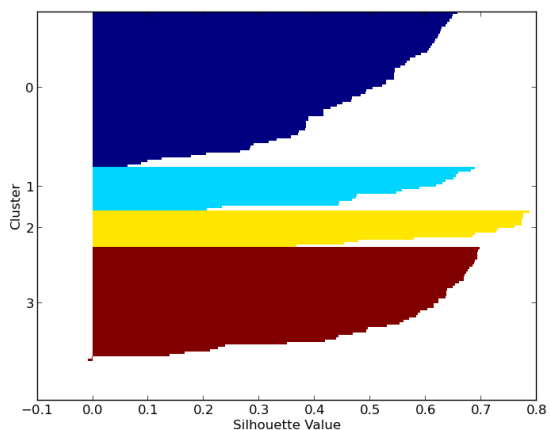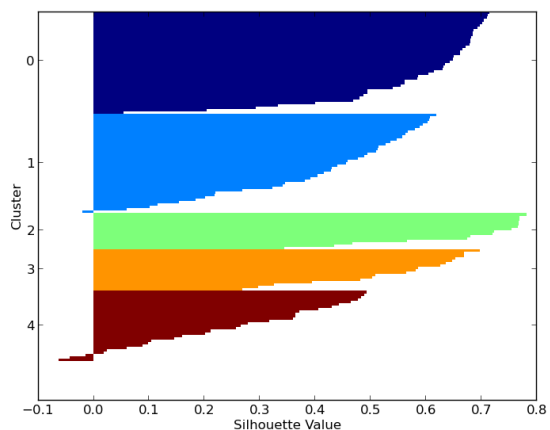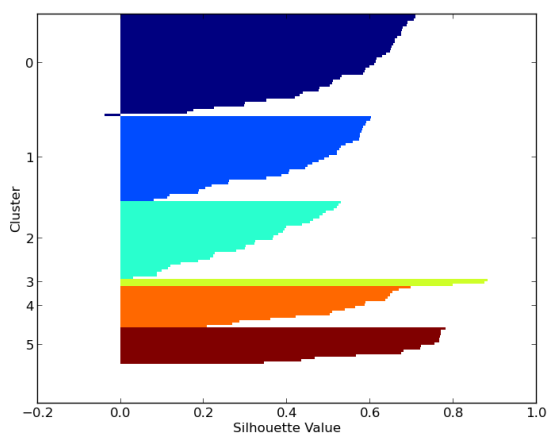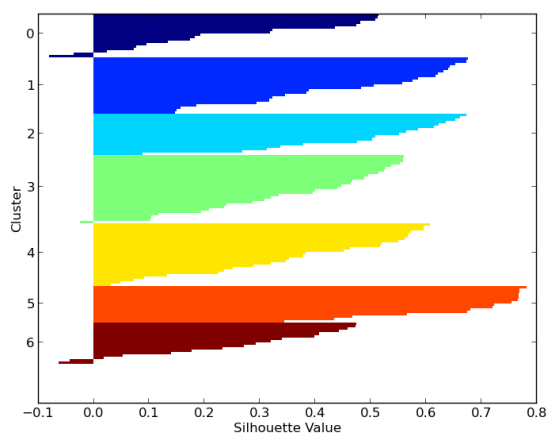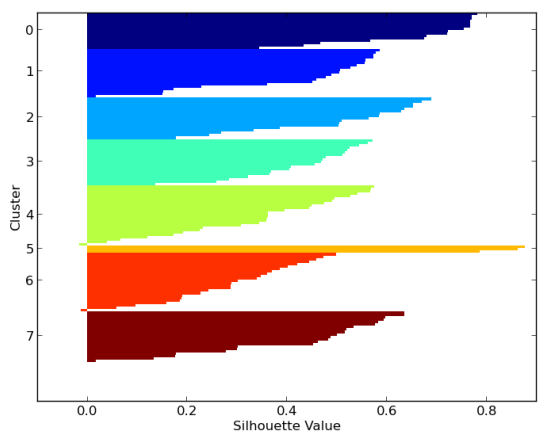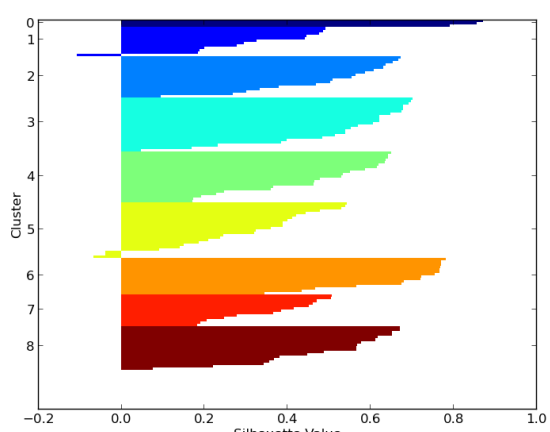

Supplement: S3 Fig — The situation with three clusters is the most homogeneous, as an indication of the most probable number of clusters. (PDF) [file pone.0190237.s007.pdf]
